# A systematic comparative and structural analysis of protein phosphorylation sites based on the mtcPTM database

**DOI:** 10.1186/gb-2007-8-5-r90

**Published:** 2007-05-23

**Authors:** José L Jiménez, Björn Hegemann, James RA Hutchins, Jan-Michael Peters, Richard Durbin

**Affiliations:** 1 Wellcome Trust Sanger Institute, Genome Campus, Hinxton, Cambridge, CB10 1SA, UK; 2 Research Institute of Molecular Pathology (IMP), Dr. Bohr-Gasse 7, 1030 Vienna, Austria

## Abstract

mtcPTM is an online repository of human and mouse phosphosites in which data are hierarchically organized to preserve biologically relevant experimental information, thus allowing straightforward comparisons of phosphorylation patterns found under different conditions. The database also contains the largest available collection of atomic models of phosphorylatable proteins. Detailed analysis of this structural dataset reveals that phosphorylation sites are found in a heterogeneous range of structural and sequence contexts. mtcPTM is available on the web .

## Abstract 2

mtcPTM is a new database of phosphorylated protein sequences and atomic models. Analysis of the phosphosites in mtcPTM showed that phosphorylation sites are found in a highly heterogeneous range of structural and sequence contexts.

## Rationale

In recent years, several sequencing projects have revealed the complete transcriptomes and proteomes for a number of organisms, including human [1,2]. The current challenge is to place this information within the dynamic context of the cell in order to elucidate how individual molecules interact to achieve the complex behavior of cellular processes, which translates into the ability of living organisms to adapt and thrive in a myriad of environments and conditions. Thus, much effort has been invested in identifying, for example, the transcription patterns of genes and the interacting partners of proteins in order to determine the connections that establish the intricate cellular pathways [3,4]. To understand these networks fully, however, we must also comprehend how their connections are regulated when the states of individual components are altered, for example by means of post-translational modifications (PTMs). It is therefore crucial to identify which proteins can be modified as well as the effect and lifetime of the PTMs.

Among PTMs, reversible protein phosphorylation is known to play a key role in regulating a variety of processes in eukaryotes, from the cell division cycle to neuronal plasticity [5,6]. The most commonly observed phosphorylations affect serine, threonine, and tyrosine residues [7,8], although phosphorylation of histidines and aspartates has also been reported (for review [9]). Protein phosphorylation is catalyzed by enzymes called protein kinases, which are usually specific for either tyrosine or serine/threonine, with few of them being able to modify all three residues indistinguishably [10-12]. The human genome encodes 518 protein kinases [13,14], and recent estimates suggest that around one-third of cellular proteins could undergo phosphorylation [15]. Despite the progress made during the past few decades, our knowledge about regulation of protein function by phosphorylation and the basis of kinase specificity remains incomplete, mainly because of lack of data. High-throughput proteomic approaches are expected to help fill this gap because they can identify large amounts of *in vivo *modified peptides (for review [16,17]).

Protein kinases catalyze the formation of a covalent bond between a phosphate group and a hydroxyl moiety of an amino-acid side chain. Because of the size and charge of the phosphate groups, their introduction could have a local, and potentially global, effect on the modified proteins. This effect may translate into modulation of protein activity, subcellular localization, half-life, and ability to interact with other molecules [8,11]. Undoubtedly, the best characterized examples of the molecular effects of phosphorylation on proteins are from high-resolution structural studies (for review [18-20]). For example, some modifications that affect residues that are part of or in the vicinity of catalytic sites and protein docking interfaces may promote or disrupt substrate binding by a combination of steric and electrostatic effects, without apparent major local structural rearrangements Histidine-containing phosphocarrier protein (HPr) [21], isocitrate dehydrogenase [22], signal transducer and activator of transcription [STAT]3B [23], STAT-1 [24], and Stage II sporulation protein (SpoII)AA/SpoIIAB [25]). On the other hand, the modifications could cause conformational changes that result either disorder-to-order transitions (glycogen phosphorylase [26,27]) or increased local flexibility if the native amino-acid packing is disrupted (protein kinase A [28,29], mitogen-activated protein kinase [30], ubiquitin-protein ligase E3 [31], and potassium channel inactivation domain [32]). However, because of technical challenges, few atomic structures of proteins are available in their phosphorylated state.

Although atomic models of the proteins in their nonphosphorylated form can provide invaluable clues that may enhance understanding of the molecular impact of modifications on proteins or allow us to predict them [18], no public resource is available that routinely stores and provides this information. Furthermore, current phosphosite databases only address the storage and display of phosphosites [33-35], disregarding the experimental context of the phosphorylation. We have developed the mtcPTM (MitoCheck's post-translational modifications) database to address these needs. The mtcPTM database is a repository of PTMs in human and mouse proteins that aims to preserve and present the experimental evidence that led to the identification of each modification. We show that the graphical display of these data allows intuitive comparisons between phosphorylation patterns from different sources or experiments. The database also contains structural information on those modified protein domains for which the actual structure, or the structure of a close homolog, is available. In addition, we have analyzed in detail this large structural collection to investigate the molecular characteristics of phosphorylatable sites in terms of solvent accessibility, secondary structure preference, and degree of conservation. We report that, in general, modified residues are in flexible/exposed regions and, although they are no more conserved than expected, they present highly variable degrees of conservation. Finally, we elaborate on those cases of phosphorylatable residues that were found buried in the structures, predicting the structural/functional effect of their modification on these proteins. As part of the MitoCheck programme, a European Union-funded project whose overall aim is to study the regulation of mitosis by phosphorylation [36], mtcPTM was originally developed for the study of differential phosphorylation in mitosis. However, its general design is readily applicable to any data, regardless of experimental source. The database is publicly available online, and experimentalists are encouraged to submit their data for storage and display.

## Results

### Handling and storage of phosphosite data

The mtcPTM database contains data retrieved from literature, protein annotations, and other databases. In the future, the database will also display phosphorylation sites that have been mapped as part of the MitoCheck project. The mtcPTM database therefore handles quite different datasets, for which the available information varies. For example, modifications retrieved from literature and protein annotation are usually recorded as individual residues, in which experimental information can only be recovered by reading the original report. By contrast, high-throughput mass spectrometry (MS) data take the form of phosphorylated positions within peptide sequences. In this case, mtcPTM preserves the experimental context of the phosphosites by grouping the MS peptides into sets according to individual experiments and assigning to each group a hierarchical data structure that summarizes the experimental information. This simple hierarchy comprises data source (for instance, a research group or programme), experimental category (for example, label describing a set of experiments that are undertaken with a combined aim), and individual experiments (data obtained from the same sample). Thus, two experiments undertaken, for example, by MitoCheck to determine the differential phosphorylation state of a protein along the cell cycle would receive the following common labels: 'MitoCheck', 'timing', and a specific label, for example interphase or mitosis.

**Figure 1:**
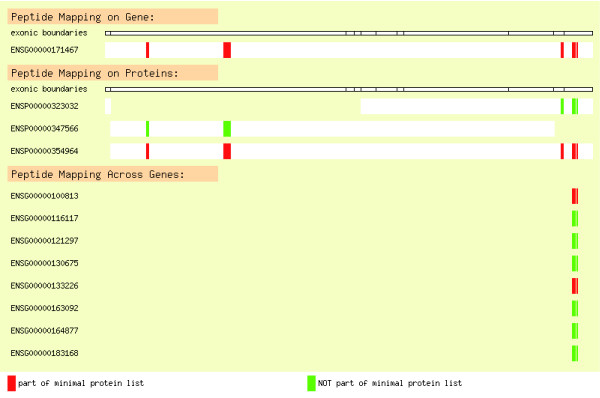
Gene view: display of genomic peptide matches. The figure depicts an example of how genomic matches of peptides from a single experiment are dealt with. Gene ENSG00000171467 (top), which has three possible transcripts/proteins (middle), was matched by several peptides obtained from an experiment. Of all the three transcripts, ENSP00000354964 was the one containing the highest number of peptides, even though none of them was unique for this protein. Therefore, ENSP00000354964 was considered to be part of the minimal list (peptides highlighted in red). However, it may be that the peptide patterns could be explained by the presence of the other two transcripts that are not included in the minimal list (peptides in green). However, even though more information would be needed to confirm either scenario, the raw data are kept for the users to draw their own conclusions. Peptides matching to proteins from other genes are shown at the bottom of the figure. Some of these protein/genes matched additional peptides and therefore they were included in the minimal list (red) whereas others did not (green). The latter assignments could thus be considered spurious.

As mentioned above, phosphosites are routinely stored as positions relative to protein sequences [33-35]. However, this has the disadvantage that if the protein entry linked to the phosphosite changes, then the information may be either lost or transferred incorrectly from one database release to the next. By contrast, storage of phosphosites as positions relative to experimentally determined, and thus invariant, peptide sequences allows their automatic update, without information loss, because the peptides can be matched regularly to the most recent version of the corresponding proteome for each new database release. The ability to update and keep track automatically of changes in the data between different releases is important not only to preserve the correct mapping of the phosphosites but also to take full advantage of improvements in genome assemblies and gene builds, especially regarding to the discrimination between splicing variants and handling of promiscuous peptides found in proteins from different genes. This is the strategy followed by the mtcPTM database. mtcPTM is based on the human and mouse genomic assemblies defined by Ensembl [37]. Each time that a new genome assembly or gene build takes place, all of the peptides stored in mtcPTM are mapped to Ensembl proteins, recording all peptide-protein and peptide-gene relationships (see Materials and methods, below). The genomic mapping of the peptides can be visualized online via the web interface of the database (Figure 1).

At present, the mtcPTM database stores 13,051 and 7,930 peptides from human and mouse, respectively, corresponding to 13,116 (serine: 9839; threonine: 2067; tyrosine: 1210) and 8,889 (serine: 6942; threonine: 1470; tyrosine: 477) phosphorylations. The human-related data comprise 3842 genes and 7753 proteins, whereas for mouse they represent 2721 genes and 3866 proteins.

### Display of protein phosphorylation data

**Figure 2:**
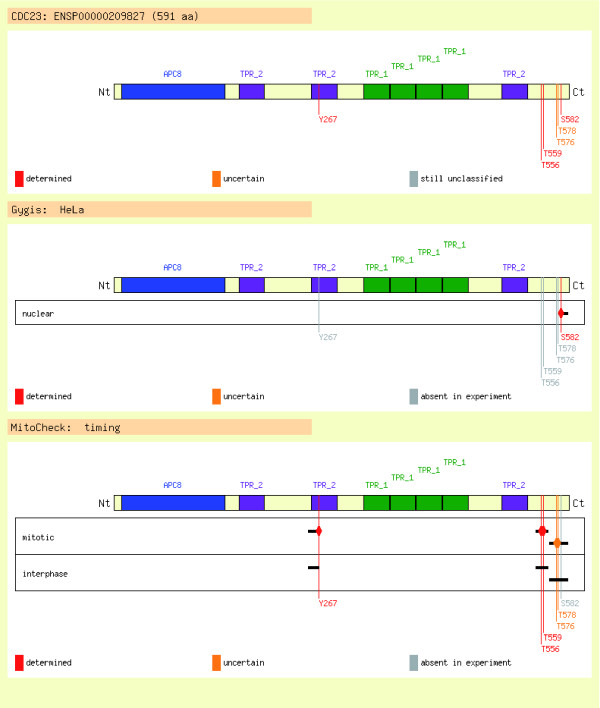
Protein view: graphical comparison of experiments. The figure shows an example of the graphical display used to present all the phosphosites stored for a given protein entry. The protein is represented by a horizontal bar, with the positions of known domains and phosphosites indicated by colored boxes and vertical lines, respectively. The top panel depicts a complete summary of all modifications, in which phosphosites are color coded according to whether they were fully resolved by the experiment, because sometimes the position of a phosphosite cannot be unambiguously determined by mass spectrometry. Thus, confidently determined positions are shown in red, uncertain positions in orange, and positions that have been retrieved from literature or other sources and are still awaiting manual curation to confirm their status in gray. The peptide maps for each experiment are then shown underneath, in which related experiments are grouped together to allow easy comparison. The color coding is the same as above with the exception that gray is now used to highlight residues that have been seen phosphorylated but not in that particular experiment. Further information about individual peptides can be retrieved via links from the lines representing them. These peptide pages include details about the sequence of the peptide, experimental data (such as protease and software used for their identification), whether the peptide is unique for a gene/protein, its position in the full-length protein, and whether there exist sequence variations with respect to the Ensembl sequence.

The website presents the data for each protein on individual pages. The tables and graphics in these pages summarize all known modifications from different experiments, along with relevant literature and information about the number and type of sequence and structural domains present in the protein as well as the frequencies of residues flanking the modified sites [38]. In particular, the comparison of the phosphorylation patterns under various conditions is implemented as a graphical display in which the experiments are grouped, according to the previously mentioned hierarchy, into different tracks where the raw data, namely (un)modified peptides, are schematically represented (Figure 2).

The database also contains structural models for proteins and protein domains that contain modified residues. These models have been automatically built by homology modeling to empirically determined atomic co-ordinates. A conservative criterion for assignment of sequences to structures was used in order to minimize errors in the modeled domains (see Materials and methods, below). The coordinates of the models are provided as RasMol scripts [39], including the pair-wise alignments between the modeled Ensembl sequences and its structural templates. The mtcPTM database currently contains 2,599 structural models, 658 for mouse proteins (comprising 529 genes), and 1,191 for human (686 genes). On comparing the phosphosite dataset with these models, only a small proportion (10% in both human and mouse) of phosphosites were found in structurally defined regions. This finding is not expected to be caused by bias resulting from the type of structural data currently available, because similar proportions were observed when counting modified positions within the far more diverse Pfam domains (85% for both human and mouse proteins fell outside defined Pfam domains) [40]. This suggested that phosphorylated sites tend to be found in flexible, unstructured segments and linkers between domains, which is in agreement with previous observations [41].

Interestingly, the distribution of residues between linkers and (structured) domains was not even. Phosphorylated threonine and serine residues were mainly located outside domains (structures). In mouse, 86% (91%) of serines and 83% (87%) of threonines were found in linkers between domains (structures), and similar numbers were obtained in human, specifically 87% (92%) serines and 83% (90%) threonines. However, this distribution was less biased for tyrosines, in which 37% (34%) in human and 31% (31%) in mouse were found within domains (structures). At present, it is unknown whether these differences between tyrosine and serine/threonine residues correlate with their propensity to appear in structured and flexible regions, respectively, or whether it actually reflects a biologically distinct feature of their regulation, such as specific properties in kinase recognition. Of note, the existence of different structural rules for substrate binding between serine/threonine and tyrosine protein kinases has previously been suggested [42].

As mentioned previously, atomic information from modified and unmodified forms of the proteins is invaluable in rationalizing the molecular effect and functional impact of phosphorylations. Therefore, even though a considerable proportion of phosphosites is situated away from structured regions, we wished to take advantage of the large structural dataset collected here to undertake a detailed study of the properties of these residues, as well as the potential effect of their modification on the domains.

### Compiling a nonredundant set of structural models

For this analysis, we first defined a nonredundant (NR) set from all of the structural models stored in the database in order to preclude potential biases arising from the comparisons of highly similar structures (see Materials and methods, below). The NR set comprised 324 structural models, representing a wide range of Pfam domains, and contained 264 modified serine/threonine and 219 tyrosine residues. Half of the models were less than 150 amino acids long, indicating that half of the models represented single domains and the other half multidomain structures. Regardless of their length, the majority of the models (72%) contained only one phosphorylated residue. Furthermore, 70% of the models shared at least 80% sequence identity with their templates and only 15% less than 40%; therefore, the overall quality of the models is expected to be high.

For the structural analyses, the phosphorylated sites were clustered into two groups: one composed of serine and threonine residues, the other of tyrosines. This grouping is based on the similar characteristics of serine and threonine, and the fact that they are usually targeted by the same protein kinases. The study focused on the following structural features of the phosphosites: relative position within structured domains, solvent accessibility, secondary structure preference, and degree of conservation.

### Phosphosites can accumulate at the flanks of structured domains

**Figure 3:**
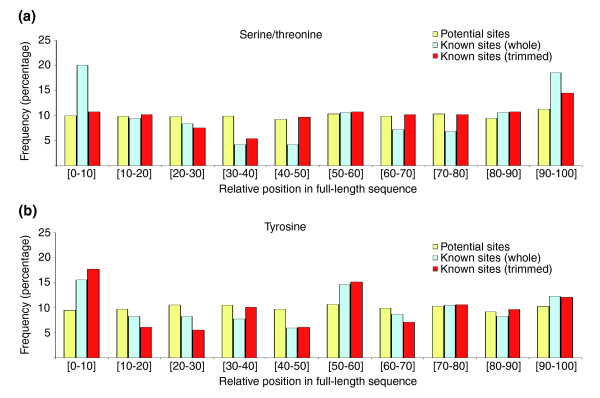
Phosphosite location relative to the structured domains. The plots show the distributions with the frequencies of occurrences of potential (yellow) and known (cyan and red) phosphosites along the length of the structures. The positions correspond to all nonoverlapping and equally long tenths in which the sequences can be split, from the amino- (left) to the carboxyl-termini (right). The distributions are shown separately for **(a) **serine/threonine and **(b) **tyrosine residues. As explained in the main text, the occurrences of known phosphosites were calculated in two different ways: directly from the full-length structure (cyan) or from trimmed versions of the domains in which disordered and exposed termini had been removed (red).

We first investigated the relative locations of phosphosites within the structures by dividing the length of the domains into 10 equally long, non-overlapping segments, and then counting the number of potential and known phosphorylated residues within each segment. This partitioning normalized differences in length between the structures. Figure 3 shows that the distribution of potential phosphorylated residues (any serine/threonine or tyrosine) in the structural models was nearly constant along the length of their sequences. Remarkably, this was not the case for known phosphosites. Phosphorylated serine/threonine residues were over-represented at both termini (Figure 3a), whereas modified tyrosines accumulated towards the amino-terminus and the middle (Figure 3b). However, this analysis did not take into account whether the terminal regions corresponded to the first (or last) structured elements of the structured domains or to the unstructured tails preceding (or following) them. The latter could have affected considerably the distributions, especially in the case of models based on nuclear magnetic resonance (NMR) structures, in which long flexible termini are sometimes reported even though they are not an integral part of the globular cores. Therefore, to account for this, all terminal residues before (after) the first (last) structured (as defined by Define Secondary Structure of Proteins [DSSP] [43]) or buried (as defined by NACCESS [44]) residue of the amino- (carboxyl-)termini were removed from the models. Thirty per cent of all serine/threonines and 10% of tyrosines were found within these tails. After removal of the disordered termini from the calculations, the distribution of serine/threonines was now closer to that expected by chance (Figure 3a). Nevertheless, tyrosine residues still seemed to be over-represented at the amino-terminus of the structured domains (Figure 3b), where nearly 50% of these terminal tyrosines were found no more than five amino-acids away from the beginning of the domains (data not shown).

**Figure 4:**
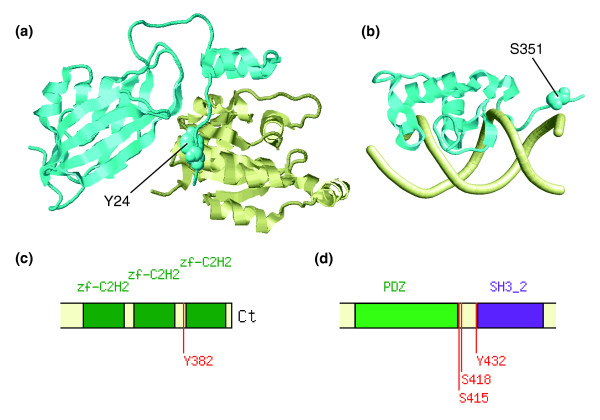
Phosphosites at unstructured termini. **(a) **Structure of the Rho GDP-dissociation inhibitor 2 in complex with RAC [81] (Protein Data Bank [PDB]: 1ds6). **(b) **Structure of the orphan nuclear receptor NR4A1 bound to DNA [82] (PDB: 1cit). In both panels the phosphosite-containing domains are colored in cyan and their interacting partners in light yellow. The modified sites are shown in space-filled representation. **(c,d) **Two examples of phosphorylations found in short linkers between domains within the human Zinc finger protein 174 and the mouse discs large homolog 4, respectively. Notice that, for the latter, the displayed boundaries of the PDZ domain correspond to those from the structural assignment and not to those defined by Pfam, because the latter did not include the carboxyl-terminus. A list with additional details on the examples, including links to the appropriate mtcPTM entries, can be found in Additional data file 1.

Closer inspection of the examples in which phosphorylated residues were found in unstructured tails flanking the core domains allowed us to group them into three different categories. The first group included termini that, although unstructured, were an important part of the interface of interaction with other molecules. Two examples of human proteins exhibiting this behavior were the Rho GDP-dissociation inhibitor 2 (ENSP00000228945) and the orphan nuclear receptor NR4A1 (ENSP00000243050). In the former, the phosphorylatable amino-terminal residue Y24 [45] was found to be tightly packed in the binding interface of the Rho GDP-dissociation inhibitor 2 with Rac (Figure 4a). In the latter, the S351 residue [46] was at the unstructured carboxyl-terminus of the domain participating in DNA-protein interactions (Figure 4b). It is known that phosphorylation of S351 in the orphan nuclear receptor NR4A1 decreases transcriptional activity by modulating DNA binding [46], and it is likely that the phosphorylation state of Rho GDP-dissociation inhibitor 2 will also modulate Rac binding.

The second group contained residues that were in short linkers joining adjacent domains. Examples of these are the human Zinc finger protein 174 (ENSP00000268655) and the mouse discs large homolog 4 (ENSMUSP00000018700). In the first example, the phosphorylation [45] can take place between two zinc-finger motifs (Figure 4c). Modifications targeting the short linkers joining zinc-finger domains were also found in other proteins (data not shown), and they may regulate oligonucleotide binding because the phosphosites are part of the putative DNA binding interface. In the second example, a number of phosphosites [47] accumulated between the PDZ and SH3 domains of the mouse discs large homolog 4 (Figure 4d), and it is tempting to speculate that the phosphorylated state of the residues may affect the relative positioning or allosteric communication between the domains.

The last group corresponded to those sites located in long and unstructured termini relatively far away from the domains. These models were mainly built from NMR structures. For these cases, it is difficult to predict the effect that the phosphorylations could have. However, by analogy to the effect observed in other examples and considering that disordered regions appear to play important roles in protein-protein recognition events [48], the phosphorylation state of these sites may regulate the interaction of additional effectors to these regions, which may be especially important for those in closer proximity to the structured domains.

### Phosphorylatable residues are not always accessible to solvent

**Figure 5:**
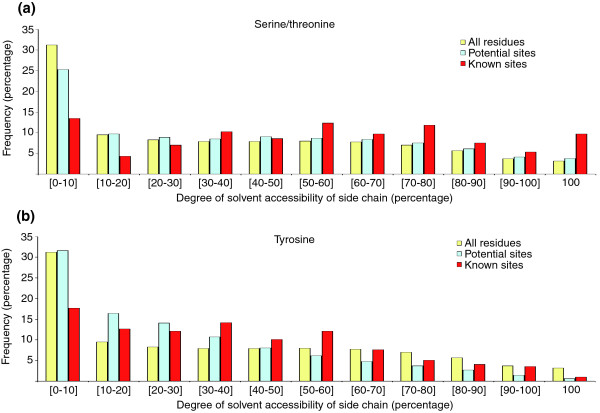
Solvent accessibility of phosphorylatable residues. The plots show the distributions of the percentage of solvent accessibility of the **(a) **serine/threonine and **(b) **tyrosine side chains in the structures, as calculated by NACCESS [44]. The cyan and red columns correspond to the distributions for all potential and known phosphorylated residues, respectively, whereas the yellow columns are controls summarizing the solvent accessibility of all amino acids. Exposed terminal regions were not included in the calculations. These distributions were identical to that calculated from the templates or from models sharing at least 80% identity to the templates, indicating that, overall, the modeled conformations of the residues holding the phosphosites are expected to be accurate.

Next, we wished to assess the accessibility of phosphorylatable residues to solvent and thus to protein kinases. Figure 5 shows the plots with the distributions of the calculated percentage of solvent accessibility for the side chains of known phosphorylated residues as compared with that of all residues and potential phosphosites (any serine/threonine or tyrosine). It is clear that the side chains of phosphorylated residues tend to be more exposed. This trend is specially pronounced for serine and threonine, which are two relatively small amino acids, and less so for tyrosine, which probably is because its large hydrophobic ring is usually at least partly protected from solvent. These results were not surprising because phosphorylatable residues would need to fit into the substrate recognition clefts of protein kinases. Therefore, it was intriguing to note that nearly 15% of all phosphosites exhibited less than 10% solvent accessibility of their side chains in the unmodified form of the protein. These buried residues would not only have problems acting as substrates for kinases, but they could also require local amino-acid re-packing to accommodate the different electrostatic and steric properties between the unmodified and phosphorylated states (see below for detailed descriptions of several examples of buried phosphosites).

### Phosphorylated serine/threonines show a marginal preference for loops, whereas tyrosines do not

**Figure 6:**
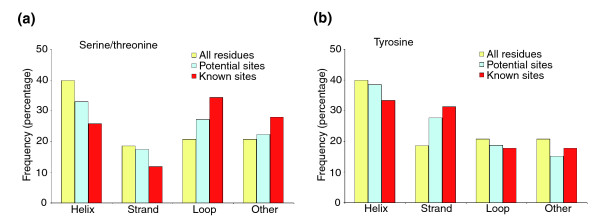
Distribution of phosphosites with respect to secondary structure elements. The plots represent the frequency of occurrences of phosphorylated **(a) **serine/threonine and **(b) **tyrosine residues in the elements of secondary structure of the models as defined by Dictionary of Protein Secondary Structure (DSSP) [43]. The three sets shown as well as their color coding are identical to those from Figure 5.

Another question to be addressed was whether phosphorylated residues exhibit any preference for particular structural elements. For this, the number of occurrences of phosphosites in four types of secondary structure elements (as defined by DSSP), namely helices, strands, loops and other, was counted excluding all terminal residues preceding (following) the first (last) structured amino acid (see above). The results are summarized in Figure 6. It appeared that phosphorylated tyrosines did not prefer any particular structural environment (*P *= 0.64) when compared with all tyrosines (Figure 6b). On the other hand, there was a marginal preference (*P *= 0.08) for phosphorylated serine/threonine residues to be located in disordered regions connecting strands or helices (Figure 6a).

### Phosphosites are not more conserved than expected

Because PTMs can play functional roles, phosphosites would be expected to be under purifying selection, and thus conserved through evolution. To investigate this, multiple sequence alignments were calculated from homologs to the modeled structures [49], and the conservation of each position corresponding to the phosphosites was assessed. The initial alignments, which can be retrieved via the mtcPTM web interface, contained nonredundant sequences sharing at least 30% sequence identity with the model. Although the inclusion of sequences that were up to 30% identical to the query domain ensured that they would adopt nearly identical structural arrangements to it [50], the alignments could present not only orthologous but also paralogous domains [51]. For the latter, the phosphorylation patterns may be different or absent because of functional divergence. Furthermore, the alignments may also contain sequences from distantly related organisms in which the phosphorylation patterns may have evolved differently. To account for these potential sources of variability, the degree of conservation of each phosphosite was assessed for several subdivisions of the initial alignments. Briefly, conservation scores were calculated for the full alignments (all sequences at least 30% identical to the query) and for three subsets containing only sequences that were at least 40%, 50%, or 60% identical to the query. In alignments obtained from sequence identity cut-offs equal to or higher than 40%, most sequences are expected to be orthologous [51].

**Figure 7:**
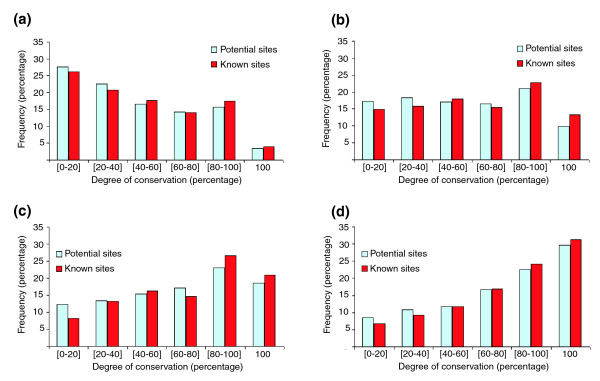
Evolutionary conservation of phosphorylated sites. The plots show the distribution of the percentage of known (red) or potential (cyan) phosphosites presenting a given degree of conservation (between 0 and 20, 20 and 40, and so on) in four sets of multiple alignments. These four sets of multiple alignments, which contain different sequence diversity, comprise sequences sharing at least **(a) **30%, **(b) **40%, **(c) **50%, or **(d) **60% identity with respect to the human or mouse queries.

The overall trends for the two-amino-acid subgroups (serine/threonine and tyrosine) were similar, and therefore the two sets were merged (Figure 7). At a low identity cut-off (>30%) very few sites were highly conserved (Figure 7a). Only less than 5% of the sites were strictly conserved across the alignments, and not more than 20% of the sites were conserved in at least 80% of all of the homologs within the alignments. As expected, the degree of conservation increased with increasing cut-off (Figure 7a to 7d). However, even for domains sharing overall sequence identities of 60% (and thus likely to contain only orthologs from closely related organisms), a considerable number of sites (about 16%) exhibited conservations lower than 40% (Figure 7d). Interestingly, in all subdivisions, the degree of conservation of known phosphosites was nearly identical to that from potential, solvent accessible, phosphosites.

### What happens when phosphorylatable sites are buried

As mentioned above, most phosphorylatable sites were considerably exposed to solvent and thus potentially accessible by protein kinases. However, for a few phosphosites, their side chains were found to present not only low solvent accessibility but to be actually packed into the domain core. Modification of these buried residues is likely to have structural implications because the intramolecular packing between the two states may be different. Depending on the amount of atomic interactions involved, the conformational changes could have local or global effects, from rigid body displacements to partial or total unfolding. In fact, our dataset contained some examples of proteins that have already been shown to undergo conformational changes upon phosphorylation (mitogen-activated protein kinase [30] and ubiquitin-protein ligase E3 [31]). In both cases the structural rearrangements are critical for activation of the proteins.

Given the intriguing nature of the buried phosphorylatable residues, we studied them systematically to elucidate how the phosphorylation could take place and what its potential structural impact could be. During the analysis, in order to ensure that the conformation of the residues of interest was likely to be native, only models in which the phosphorylatable side chains had been built based on the same or similar residues from the templates were considered. We also checked the consistency of poor solvent accessibility for those residues in which there existed other available models, with similar sequence identity to the templates, in the redundant set. We found 13 examples of this in which ten exhibited similar low accessibility (at a 10% cut-off) and three examples in which both the exposed and buried versions could exist, depending on the conformational states of the proteins. The latter included the active and auto-inhibitory conformations of human tyrosine-protein kinase c-Src [52,53]. The other two examples are discussed below.

The analysis of phosphorylatable buried residues revealed that their modifications could have three major structural/functional effects on the structures: regulation of function by affecting functional sites directly or indirectly; spatial rearrangements, presumably by rigid body movements, of domains within a protein; and opening of the structure, leading to local flexibility.

### Phosphorylation of buried residues found at or close to functional sites

**Figure 8:**
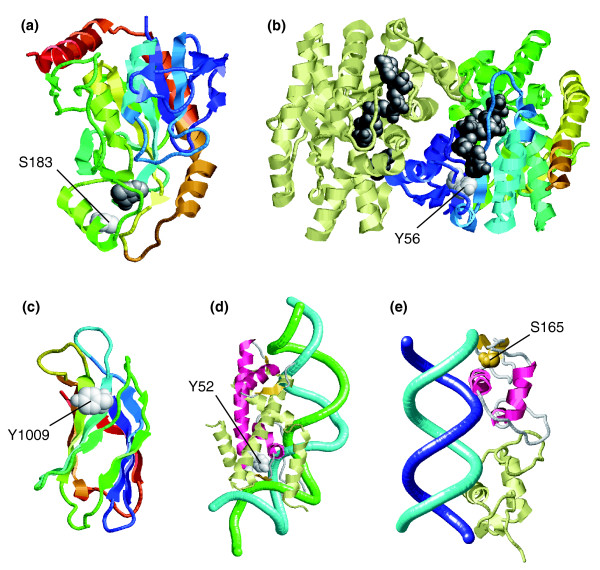
Phosphorylatable buried residues at or close to functional sites. The figure shows examples of phosphorylatable buried residues that are found at or in the vicinity of binding sites for small or large molecules. All the structures are shown differentially colored from their amino- (cold colors) to their carboxyl-termini (hot colors), with the phosphosites in white space-filled representation and their bound substrate in dark gray unless stated otherwise. **(a) **Structure of human 5'-deoxy-5'-methylthioadenosine phosphorylase [83] (Protein Data Bank [PDB]: 1cb0) with an adenine molecule. **(b) **Structure of homodimeric human malate dehydrogenase type 2 bound to NAD co-factor (unpublished data; PDB: 2dfd). **(c) **Structure of the carboxyl-terminal C2 domain of human tricalbin modeled onto the C2 domain of phospatidylinositide 3-kinase C2 α [55] (PDB: 2b3r). **(d) **Structure of a histone dimer (red and light yellow) bound to DNA (green and cyan) [84] (PDB: 1kx5). **(e) **Structure of the homodimer formed by the zinc-finger domains of the estrogen receptor in complex with DNA (cyan and blue) [85] (PDB: 1hcq). A list with additional details of the examples, including links to the appropriate mtcPTM entries, can be found in Additional data file 2.

Active sites and binding pockets for small/medium-size molecules are usually inside clefts. Therefore, phosphosites found around them are likely to be, at least partially, buried. Their phosphorylation may affect either directly or indirectly the integrity of the functional sites depending on whether they are part or in the vicinity of them, respectively. An example of the latter can be found in human 5'-deoxy-5'-methylthioadenosine phosphorylase (ENSP00000369519), where the buried phosphosite S183 [54] sits in a peripheral helix that is part of the binding groove for adenine (Figure 8a). On the other hand, an example of a phosphorylatable residue [45] that is actually involved in substrate binding can be seen in human malate dehydrogenase type 2 (ENSP00000327070), in which the nonphosphorylated version of the residue Y56 interacts with the NAD co-factor (Figure 8b).

It is tempting to hypothesize that phosphorylation of the carboxyl-terminal C2 domain of human tricalbin (ENSP00000267113) could also regulate its substrate-binding capability. This domain is phosphorylated at position Y1009 [45], which is located at the concave face of the β-sandwich, a region rich in positively charged residues (Figure 8c). In homologous domains, including the template used for the modeling [55], this poly-basic region can have phospholipid-binding capabilities [56]. Therefore, covalent attachment of a phosphate to this tyrosine would alter the net charge of the region and thus could affect its putative ability to interact with lipids. Of note, the equivalent tyrosine (Y822) in another C2 domain within this protein can also be phosphorylated [45]. Interestingly, these tyrosines are very conserved across C2 domains, including distantly related paralogs. However, phosphorylations at these positions have not been reported for other well characterized C2 domain subfamilies. Therefore, phosphorylation of this tyrosine may be an exclusive feature of some tricalbin C2 domains.

In addition to phosphosites in binding pockets for small substrates, modifications were also found in areas involved in binding to larger molecules. This was the case for histones (ENSP00000350159) and the zinc-finger domain of the human estrogen receptor (ENSP00000343925). In the latter, the residue that can be phosphorylated, namely S165, is found at a short loop, which is in direct contact with DNA (Figure 8e). Phosphorylation at S165 is critical for activation of the transcription factor, perhaps by precluding interactions with inhibitors that may occlude the DNA-binding interface [57]. In the histone, the phosphorylatable Y52 residue [45] is packed into one of the two small cores of the histone domain (Figure 8d). This region is involved in both DNA binding and interactions with another histone monomer. In this case, the introduction of phosphate groups may also regulate the DNA-binding or protein-binding capabilities of the domains.

### Phosphorylation of residues buried between domains within the same protein

**Figure 9:**
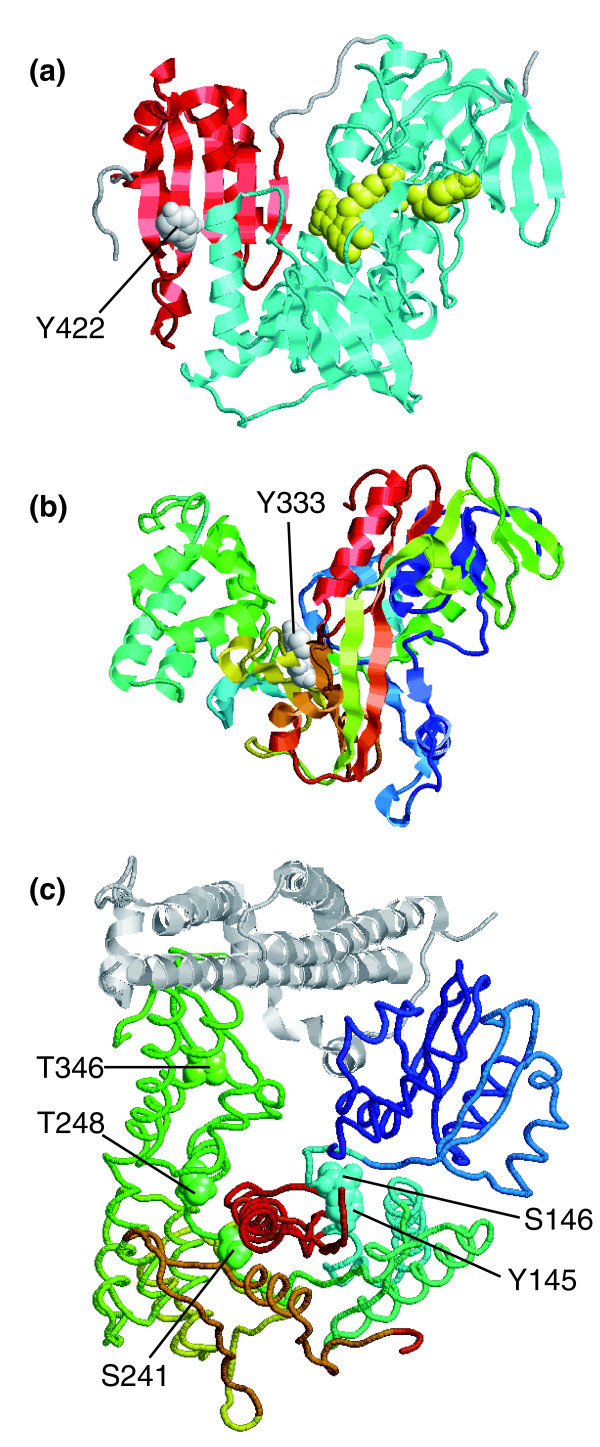
Phosphorylatable residues buried between domains. The figure shows examples of phosphorylated buried residues found in hinge regions or at the interface between domains from the same protein. All of the structures are shown differentially colored from their amino- (cold colors) to their carboxyl-termini (hot colors) and the phosphosites in white space-filled representations unless stated otherwise. **(a) **Structure of human thioredoxin reductase 1 (unpublished data; Protein Data Bank [PDB]: 2cfy). The residue that can be phosphorylated, Y422, participates in the interface between the two domains of the protein, namely the pyridine nucleotide-disulphide oxidoreductase (cyan) and the dimerization (red) domains. The substrate for the enzyme is shown in yellow. **(b) **Structure of the α isoform of the guanine nucleotide dissociation inhibitor [86] (PDB: 1gnd). **(c) **Structure of mouse Sec1 complexed with syntaxin-1 (light gray) [58] (PDB: 1dn1). The phosphorylatable residues are shown in the same color as the protein backbone to facilitate their localization along the structure. A list with additional details of the examples, including links to the appropriate mtcPTM entries, can be found in Additional data file 2.

When buried residues are found in hinge regions or at the interface between domains within the same protein, their modification could trigger changes, for example, by means of rigid body rotations or translations, in the relative positioning between domains in order to accommodate the new covalently attached phosphates. Examples of phosphosites packing at the interface between domains were found in human thioredoxin reductase 1 (ENSP00000373506; Figure 9a) and the α isoform of the human guanine nucleotide dissociation inhibitor (ENSP00000369538; Figure 9b). On the other hand, examples of phosphosites at potential hinge regions between domains were present in mouse Sec1 (ENSMUSP00000052440). Figure 9c shows that its buried, phosphorylatable residues [47] pack at the interfaces between domains (Y145, S146, S241, and T248) or at the domain core (T346). Interestingly, the residues affected are important for the maintenance of the U-shaped conformation that recognizes syntaxin-1. Therefore, their modification, along with that of other surface residues [58], may have functional implications regarding the ability of Sec-1 to bind syntaxin-1. All of these residues were relatively well conserved except for Y145. However, the latter was otherwise replaced by phenylalanine, suggesting that this position does indeed play an important structural role in domain packing.

### The phosphorylation state of buried residues could influence the structural conformation of the protein

When buried residues that participate in the packing of the protein core or of isolated secondary structure elements to the globular domain can be phosphorylated, their modifications could lead to structural instability. In the case of isolated elements of secondary structure, for example those at the domain termini, this instability may lead to their detachment, perhaps without compromising the structural integrity of the core domain. On the other hand, when the modified residues are part of the hydrophobic core of the domain, the modification could cause considerable structural rearrangements, including local or even total unfolding, if the packing of the unmodified residue were critical for the maintenance of the overall structure and no alternative stable packing for its phosphorylated form could be established.

**Figure 10:**
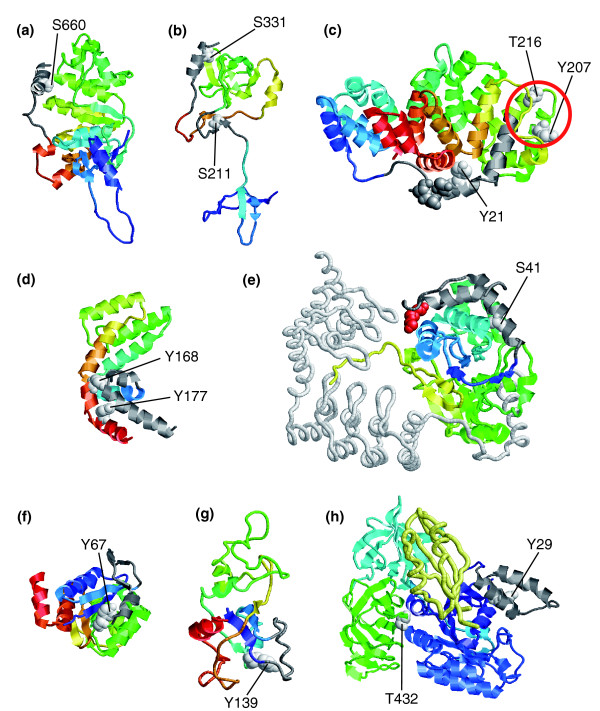
Buried residues whose phosphorylation state could affect local structural conformation. The figure shows several examples of buried residues whose phosphorylation may result in conformational rearrangements, including detachment of secondary structure elements from the protein domain. Unless stated otherwise, all the structures are shown differentially colored from their amino- (cold colors) to their carboxyl-termini (hot colors), with the regions whose conformation is predicted to be affected in gray, and the phosphosites in white space-filled representation. **(a) **Structure of the human 7508A NBD1 domain [60] (Protein Data Bank [PDB]: 1xmi). **(b) **Structure of the autoinhibited p47^phox ^[62] (PDB: 1ng2). **(c) **Structure of annexin-1 [64] (PDB: 1hm6). Other residues, namely T24, S27 and S28, that can also be phosphorylated, although they are not buried, are shown in gray. The region encircled is likely to be affected by phosphorylation of the enclosed amino acids, as described in the main text. **(d) **Structure of the regulator of G-protein signaling 16 (unpublished data; PDB: 2bt2). **(e) **Structure of the serine/threonine protein phosphatase PP1-β catalytic subunit [67] (PDB: 1s70). A PEG molecule is shown in red and the 130 kDa myosin-binding subunit of smooth muscle myosin phosphatase in white. **(f) **Structure of DJ-1, a protein related to male fertility and Parkinson's disease [69] (PDB: 1ps4). **(g) **Structure of the 60S ribosomal protein L7-A [70] (PDB: 1s1i). **(h) **Structure of the elongation factor EEF1A [87] (PDB: 1f60), in which their individual domains are shown in blue (elongation factor Tu GTP-binding domain), cyan (elongation factor Tu domain 2), and green (elongation factor Tu carboxyl-terminal domain). The catalytic carboxyl-terminal domain of EEF1BA is shown in yellow. A list with additional details of the examples, including links to mtcPTM entries, can be found in Additional data file 2.

There were several examples of buried phosphorylatable residues located at terminal structural elements. Three of these examples have already been proposed to alter the conformation of the affected proteins. The first example was the human 7508A NBD1 domain (ENSP00000003084), in which the carboxyl-terminal helix of the first ABC transporter domain contains a buried serine residue (S660) that can be phosphorylated [59]. The residue participates in the packing of the helix to the domain (Figure 10a). The helix and its preceding loop are rather flexible and their conformational preference may be altered by phosphorylation of S660 [60]. A second example was the auto-inhibited human p47^phox ^(ENSP00000297905), in which the carboxyl-terminal helix packs to a SH3 domain via a phosphorylatable serine at position 331 (Figure 10b) [61]. Additional phosphorylation sites are found in another buried residue, namely S211 (Figure 10b), as well as several exposed serines (306, 307, 318, and 323) at the loop preceding the carboxyl-terminal helix. Multiple phosphorylations involving these sites have been seen to unmask the SH3 domain, facilitating interactions with other proteins that ultimately result in the formation of an active enzyme complex [62]. It is worth noting that the conformation of these domains is very different when complexed to a p22^phox^-derived peptide [63], where the S211 residue does not pack intramolecularly. The third example was the structure of human annexin-1 (ENSP00000257497). This protein has three buried residues that can potentially be phosphorylated (Figure 10c): Y21, Y207, and T216. Y21 is found after the first amino-terminal helix packing against the globular part of the protein, whereas Y207 and T216 are both part of a peripheral two-helix bundle. It has been suggested that multisite phosphorylation of Y21 and neighboring residues (T24, S27, and S28) (Figure 10c) exposes the amino-terminus, which is critical for vesicle aggregation [64]. This terminal region is specific for different annexins and presents intrinsic flexibility [64]. In the region comprising Y207 and T216, we found that T216 also packs onto a flexible loop as deduced from its high temperature factor in the crystal structure and that the solvent accessibility of Y207 slightly varies in other crystal structures due to the different conformation of the neighboring arginine 212 [65]. Furthermore, T207 and T216 are both surrounded by charged residues, and thus their phosphorylation could induce electrostatic- and steric-induced rearrangements.

Additional examples of phosphorylatable buried residues at terminal regions were found in the regulator of G-protein signaling 16 and the serine/threonine protein phosphatase PP1-β catalytic subunit. In the former (ENSP00000356529), the phosphorylation of Y177 [66], which is found in the carboxyl-terminal helix packing tightly against the amino-terminal helix (Figure 10d), may disrupt the interaction between these two helices. This effect could be reinforced by additional phosphorylation of Y168, which also packs against the carboxyl-terminus of the amino-terminal helix (Figure 10d). Both tyrosines are well conserved and only occasionally replaced by phenylalanines, which is indicative of their important structural roles. In the human serine/threonine protein phosphatase PP1-β catalytic subunit (ENSP00000351298), S41 [54] is found in the second amino-terminal helix (Figure 10e), whose packing to the domain appears critical in maintaining an optimal arrangement of the two terminal helices. In the crystal structure, the loop joining these two helices participates in the binding to a PEG molecule and, to a lesser extent, to the 130 kDa myosin-binding subunit of smooth muscle myosin phosphatase [67]. Of note, S41 is only conserved in closely related sequences and is otherwise replaced by small hydrophobic residues that could still allow the helix to pack against the domain.

There were also examples of buried residues in nonterminal regions for which their phosphorylation may induce local structural rearrangements. The first example was the structure of human DJ-1, a protein that is related to male fertility and Parkinson's disease (ENSP00000340278) [68]. Y67 is found in a loop that contributes to the packing of a short helix against the domain (Figure 10f). This helix is the most disordered region in the electron density of the crystal structure [69]. Its flexibility could increase upon phosphorylation of Y67, because the phosphorylated amino acid may preclude its packing to the domain. This could ultimately affect the preceding loop, which participates in the dimer interface in the crystal structure. Interestingly, Y67 is poorly conserved, although the important role of this position is evident because the preferred replacement is phenylalanine. The second example was the human 60S ribosomal protein L7-A (ENSP00000339795). Here, Y139 packs against a helical motif on the surface of the protein (Figure 10g). Its phosphorylation may affect the conformation of this motif, which is involved in RNA-protein interactions [70]. Finally, the structure of the human elongation factor EEF1A was an excellent example of both potential local conformational rearrangement and domain re-orientation (ENSP00000339053). The first involves Y29 (Figure 10h), which is found packing in the core of a helical subdomain formed at the amino-terminal elongation factor Tu GTP-binding domain. The second involves T432 (Figure 10h), which is packed between two domains, namely elongation factor Tu GTP-binding domain and elongation factor Tu carboxyl-terminal domain, of this multi-domain protein. Phosphorylation of T432 may affect the relative orientations between domains within the protein, whereas Y29 may modulate the conformation of the helical subdomain. The orientations of the domains and of the helical bundle are likely to be critical for an effective interaction of the protein with the catalytical carboxyl-terminal domain of EEF1BA (Figure 10h).

## Discussion

We have presented a database, mtcPTM, that stores human and mouse phosphosites [71]. The database integrates data from low-throughput and high-throughput screenings and, in contrast to other, similar databases, it explicitly preserves the experimental context for each phosphosite by means of a three-level hierarchy annotation. Furthermore, the phosphosites are stored as relative positions within experimentally determined peptides, which allows automatic updates without loss of information, against new genome assemblies or gene builds. The data are publicly accessible via a web interface, in which the user can retrieve the mapping of the peptides onto the Ensembl genome as well as extensive information about the phosphorylated proteins including graphical comparisons of phosphorylation patterns under different conditions. At present, only mass spectrometric data are explicitly referenced to their experimental sources. In the future, the low-throughput data could be integrated likewise by, for example, using the relevant literature references to manually assign the data hierarchy.

Another major asset of the mtcPTM database is that it contains atomic models for a considerable number of phosphosites. These models have been automatically built by homology to experimentally determined structures using a conservative procedure in order to minimize modeling errors. Although a higher number of structural models could have been obtained by the use of more sensitive fold recognition techniques, the use of templates sharing low sequence similarity with the targets may decrease the overall quality of the models. To our knowledge, the structural set provided here is the largest freely available collection of phosphorylatable proteins.

From the study of this large structural collection, some general trends have been observed. For example, we find that some termini or regions close to the termini of structured domains may be preferentially phosphorylated. These phosphosites would be readily accessible by protein kinases, and their behavior could be similar to those found in the most frequent case of unstructured regions linking domains. Also, the degree of conservation of phosphosites varies considerably from protein to protein not only with respect to distantly related species and paralogs but also between closely related organisms. Few cases of highly conserved sites were found across alignments containing very diverse sequences, and some sites were also poorly conserved even in alignments of highly similar sequences. Overall, this indicates that the evolution of phosphosites could be less constrained than that of other functional motifs, such as catalytic sites. This variation could be organism-specific for a number of cellular processes or it may reflect the different importance of phosphorylation as a regulatory strategy between organisms. Alternatively, the precise position of phosphosites may in some cases not be critical for its action, especially in proteins regulated by multisite phosphorylation [8]. Furthermore, we have also noticed that high conservation does not necessarily indicate that a given site will be phosphorylated in all homologs (for example, the C2 domains of tricalbin). Further studies will be needed to clarify and elaborate on these observations.

We have also reported that the side chains of phosphorylatable amino acids are usually exposed to solvent. However, we found a significant number of sites that were buried. It could be possible that some of these cases were artifactual, caused by experimental *in vitro *phosphorylation of non-full-length constructs, ambiguous peptide-protein assignment, or modeling artefacts. However, in most cases the models were built from structures that share high similarity to the target sequences and thus, except in regions with intrinsic high flexibility or poor steric constraints, the conformation of the modeled side chains are probably nearly native, especially for conserved buried residues. Also, all instances considered here in which there was available structural information for a phosphopeptide matching to different genes (mainly genes with multiple, highly similar copies such as histones, elongation factors and ribosomal proteins), the sequences of the modeled domains were nearly, if not completely, identical and therefore the equivalent phosphosites presented similar low solvent accessibility because they were built based on the same structural templates. Furthermore, some of these buried phosphosites were either identified under *in vivo *conditions or have already been empirically characterized in some detail, suggesting that most buried phosphorylatable residues reported here are likely to be *bona fide *phosphosites.

By exploring systematically the examples of phosphorylatable buried sites, we have been able to predict the functional impact of the modifications to some proteins and to classify the residues into three categories according to their predicted effect. These categories are residues in or close to binding pockets or intermolecular interfaces; residues at interfaces or in hinge regions between structural units of multidomain proteins; and residues whose phosphorylated state is likely to result in conformational rearrangements, including detachment of secondary structure elements. In the first two cases, the disturbance of binding sites or interfaces would introduce steric and electrostatic constraints for intermolecular binding leading to loss of activity and rigid body rearrangements between the structural units of the multidomain proteins, respectively. For the third category, it is tempting to suggest that isolated elements of secondary structure joined to the main domain by long disordered, usually loosely packed, linkers could be released to solvent upon modification of residues that pack them against the main domain. This release could serve to re-orient domains within the protein. Alternatively, the now flexible fragments or newly exposed areas on the core domain could play a role in the downstream recruitment of additional effectors. We have also observed cases in which the modifications are likely to result in local unfolding/refolding. However, we have not observed cases in which the modification could lead to major unfolding, perhaps because of the limited size of our dataset or because these modifications are rare. In any case, it is likely that these modifications could regulate the half-life of the protein.

How protein kinases could have access to the buried residues remains to be addressed. However, several possible mechanisms could facilitate kinase accessibility. If residues to be phosphorylated were in relatively loose or in disordered structural elements, then this intrinsic flexibility may result in spontaneous opening of the regions, temporarily exposing the residues to protein kinases. We have seen examples of phosphosites sitting in naturally flexible regions as identified by examining the temperature factor of the crystal structure templates (also see [72]) or the variability in the ensembles of various atomic models solved by NMR. However, it must be remembered that in some cases additional co-factors/effectors may be required to actively extract and present the residues to the phosphorylating enzymes.

Finally, although this structural study has revealed some general features, it has also made it clear that the impact of phosphorylations will depend on the atomic environment of the modified residue and thus is likely to be case specific. Therefore, we believe that making these structural data publicly available via the mtcPTM database will be of great importance to experimentalists who wish to examine in detail their particular proteins of interest. This will also provide access to the numerous examples that have not been dealt with here, including exposed functionally relevant phosphosites and those that could be silent (nonfunctional) phosphorylations.

## Conclusion

We have implemented a database of phosphorylated residues that allows straightforward comparison of phosphorylation patterns obtained from different experiments. In addition, clues about the molecular effect of phosphorylation on some proteins domains have been obtained by examining their structural models stored on the database. It is hoped that the mtcPTM database will serve as a working tool for experimentalists, but it should also be a useful resource to theoretical biologists because it houses a large collection of protein phosphorylation data. Ultimately, the knowledge obtained from mining this database will be important to further our understanding of the regulation of protein function by PTMs. Researchers are encouraged to submit their data for storage and public display.

## Materials and methods

### Genomic peptide matching

The genomic matching was performed separately for each set of peptides from individual experiments. First, the peptides were compared as strings for perfect matches to Ensembl proteins. Unmatched peptides were then compared against all Uniprot entries from a given organism [73]. When perfect matches to Uniprot were found, the matched Uniprot entry was used to extend the peptide sequences by 10 residues at both termini. The extended peptides were then BLASTed against Ensembl proteins [74]. This sequence extension was important to improve the signal-to-noise ratio of the BLAST searches, especially for short peptides, and to increase the chance of identifying the variation that precluded a perfect match, especially when the variation took place at the ends or involved insertion/deletions that could have truncated the reported alignment. Only matches covering the initial, unextended peptides were considered further.

The adequacy of the matches was then assessed by their length coverage, sequence identity and the existence of previous perfect matches to the same proteins. The philosophy of the assessment was initially to weight more favorably high sequence similarity between genomic regions and experimentally determined peptides, and only to consider low similarity cases that involved proteins previously matched perfectly by another peptide from the same experiment. Thus, this assessment had three hierarchical levels of stringency against which all of the matches for a given peptide were examined until a match was considered to be adequate. First, if the number of mismatches was zero and the length covered was at least 85% of the peptide, or the full-length peptide was matched with a sequence identity of at least 85%, then the peptide-protein match was accepted independently on whether there existed previous matches for that protein. These 85% cut-offs minimized spurious matches without losing many real ones, as deduced from a test dataset (data not shown). If no adequate peptide matches were found, then the second assessment would admit matches with values of at least 85% for both length coverage and sequence identity but only involving proteins previously identified. In the final and least strict assignment, protein-peptide matches were accepted if the protein had previously been identified and the matches had either 85% length coverage or sequence identity. At present, the peptide matching procedure does not penalize disagreements between the flanking sequences from the proteins matched by the peptides with respect to those expected by the characteristic digestion pattern of the protease employed in the experiment.

Phosphorylated sites obtained from literature and other resources were handled in a similar way because they were actually stored in the database as artificially long peptide sequences, extracted from their original source entries, with the modified residues situated at their central position. This facilitates the complete reassignment of all protein-peptide matches every time that a new version of Ensembl is released.

### Clustering peptide-protein matches from each experiment into minimal protein lists

Following the matching of peptides to genes, it was necessary to analyze further those peptides with multiple matches, either to more than one protein from a single gene or to proteins from different genes, in order to determine whether the assignment could be further restricted to a single peptide-protein or peptide-gene pair, respectively. Thus, after the initial assignment of every peptide to one or more proteins, the peptides from an experiment were grouped according to the matched proteins in order to find a minimal list of proteins that could explain all of the matches observed [75]. A protein was regarded as part of the minimal list if at least one of its peptides was unique or if it represented a unique combination of peptides that was not a subgroup of the peptide set of another protein. When the experiment provides high sequence coverage for each protein, this assignment can be unambiguous. However, when the sequence coverage is low, as is sometimes the case for high-throughput data, it can be difficult to distinguish between splicing variants or cross-matches. Because of this, even though every matched protein will eventually be described as being part or not of a minimal list, the mtcPTM database still keeps all the matches originally found, which can be visualized online for further critical inspection (Figure 1).

### Structural modeling of protein domains containing phosphorylatable residues

Only proteins belonging to the minimal lists were considered for homology modeling. The feasibility of building models for these proteins, or for fragments of them, was determined according to their significant sequence similarity to available high-resolution structures. The sequence set of determined structures was obtained from Protein Data Bank (PDB) co-ordinates [76]. Positions for which no electron density was available were given the amino acid letter code 'X'. The final set included a set of redundant sequences containing all the full-length sequences from PDB structures as well as those of their individual domains as defined by Structural Classification of Proteins (SCOP) [77,78]. This redundancy was necessary because our fold recognition procedure mandated that the regions matched onto the protein queries should cover the entire length of the structural units in order to avoid missing structural elements in the three-dimensional models.

The structural assignment was performed as follows. Each protein sequence was first BLASTed against the structural database. Matches were initially considered adequate if they covered 80% of the length of the structural unit sharing no less than 30% sequence identity. Subsequently, the regions matched on the protein were extended in both directions to account for the full-length of the structure, and both sequences were realigned using ClustalX [79]. The extension/alignment procedure was iterated until the protein covered exactly the entire length of the structure. After convergence, all pair-wise alignments with less than 30% sequence identity (including gaps) or more than 5% gapped regions in both sequences were discarded. If two possible models from the same protein covered exactly the same set of modified residues, only that with the highest sequence identity to their structural template was considered. The whole procedure was automatic and therefore the pair-wise alignments were not inspected or adjusted manually. However, given the strict sequence similarity and length coverage cut-offs imposed, it is likely that most alignments will be correct. The homology models were built from the pair-wise alignments using MODELLER with default values [80].

### Creating a nonredundant set of structural models

To make a nonredundant set of structural models the sequences of all the modeled structures were clustered using BLASTclust with cut-offs of 40% sequence similarity and 80% length coverage. After the clustering, the member with the highest number of phosphorylated sites (or highest sequence identity to the template if their number of phosphosites was identical) from each group was taken. The nonredundant set used in the structural analysis (and provided in the Additional data files) was based on the mtcPTM release corresponding to Ensembl version 40. Updated versions of the structural models can be found in the live site.

## Additional data files

The following additional data are available with the online version of the paper. Additional data file 1 provides examples of phosphosites found at the flanks of structured domains. Additional data file 2 provides examples of phosphorylatable buried residues.

## Supplementary Material

Additional data file 1 The table provides further information about the modeling as well as mtcPTM links for each protein described in Figure 4. For each entry, the columns represent the corresponding Figure number (FIGURE), Ensembl identifier (PID), link to mtcPTM (mtcPTM link), start (Nt) and end (Ct) of the structure with respect to the full-length sequence, PDB template used for the modeling (PDBID) as well as the pair-wise sequence identity (IDENT), the position of the phosphosite (RESIDUE), experimental source (EXPERIMENT), the termini involved (TAIL), and the distance to the structured domain (DISTANCE). Click here for file

Additional data file 2 The table provides further information about the modelling as well as mtcPTM links for each protein described in Figures 8 to 10. For each entry, the columns represent the same fields described in Additional data file 1, along with the addition of two new columns containing the percentage of solvent accessibility for the whole side chain (SA-side) or only for its polar atoms (SA-polar). Click here for file
